# Case of Fractured Skull

**Published:** 1827-09

**Authors:** G. Yeates Hunter

**Affiliations:** Surgeon, Margate.


*Case of Fractured Skull.* Communicated by G. YEATES HUNTER, Esq. Surgeon, Margate.

To the facilities afforded for the diffusion of rare and interesting cases, and their treatment, by medical periodical Journals, is, in my opinion, to be attributed the rapid improvement which has taken place in the different branches of the profession. From treatises and class-books written upon general principles, although highly useful for their arrangement of diseases, the young practitioner will obtain but little practical advantage; but, from a close observance of nature and her resources, and from the perusal of individual cases whose symptoms and treatment are accurately described, his knowledge will be certainly increased. With these views it is that I send you the following case, which, as it affords an instance of recovery from an unusually severe injury of the brain and head, you may perhaps think deserving of record in your valuable and widely-circulated Journal.

On Saturday, June 11th, 1825, I was called to Kingsgate, in consultation with Dr. EDWARD SCUDAMORE and Mr. KETCHLY, a respectable surgeon of Broadstairs, upon the case of Lewis Wales, a labourer, whose skull had been dreadfully fractured that afternoon, in the following manner:—He was employed in digging a well, and, when at the depth of about forty feet, while the men above were drawing up a basket of chalk, the winch broke away from the staples which fastened it to the uprights, and fell upon the poor fellow's head, from which the spindle glanced off obliquely, but the edge of the winch cut through the integuments, and produced a severe fracture of the skull. His companions immediately drew him up; and, when I saw him, his pulse was small, frequent, and intermitting; his pupils were fully dilated; his breathing stertorous; his face pallid, and bedewed with cold clammy perspiration. He had hemiplegia of the left side, and his head presented the following appearances:—The integuments were divided to the extent of four inches; the posterior superior portion of the right parietal bone was fractured and depressed; the coverings of the brain were lacerated and hanging out between the bones, and about a table-spoonful of brain had escaped. The depressed portion of bone measured two inches long, and rather more than one wide, was beaten into the substance of the brain, and could not be removed, as the inner table of the bone was broken off beyond the outer, and prevented its exit. The operation of trepanning was therefore necessary, which I immediately commenced performing by cutting through the integuments, beginning at the middle of the lower edge of the wound, so as to form the letter T; and, after sawing out two circular pieces of bone, room was afforded for the removal of the detached piece.

The fracture began an inch from the occipital, and ran parallel with the sagittal suture, about three-quarters of an inch from it. Several small pieces of bone were picked out from the substance of the brain. I cut off the lacerated membranes with my scissors, and, after smoothing the rough edges of the bones, I brought the integuments in contact by means of adhesive straps. There had been considerable effusion of blood before, but very little was lost during or after the operation.

The patient was immediately put to bed: his breathing became less laboured, and his pulse full and hard. Mr. Ketchly therefore bled him to twenty ounces, and the following mixture was directed:—

℞. Magnesiæ Sulphat. ℥j.; Ant. Tart. gr. j; Aquæ Menthæ, ℥ vj. sumat quartem partem sextis horis.

June 12th.—The bowels have been well opened, and urine voided. He has passed a very restless night, and is apprehensive of death. Violent throbbing of and pain in the head; pupils contract when light is thrown upon them; pulse ninety, full and hard; countenance less pallid, but anxious; skin hot and dry; tongue furred and rough.

V.S. ad ℥ xvj. Repet^r Mistura.—And in the evening he was again bled to ℥ xvj.

13th.—No sleep in the night; great anxiety and restlessness; pulse hard, vibrating, and eighty; bowels and bladder emptied; pain in the head still distressing. Dressings removed; wound looks favourably.

V.S. ad ℥xvj. Repet^r Mistura.

In the evening, his restlessness increased, and Dr. Scudamore, being summoned to see him, prescribed—

℞. Ext. Hyosciami ℈j.; Pulv. Ipecac. comp. ℈ij. M. et divid. in pilulas No. xij. capiat ij. hora somni.

14th.—He has had some refreshing sleep, and his mind is more tranquil, having been engaged in prayer last night with the Rev. Mr. Peel. Pulse seventy, less hard and vibrating; countenance looks better; bowels and bladder freely emptied.

Repet^r Mistura et Pilulæ.

15th.—Has had some sleep; continues calm. Hemiplegia the same. Bowels open, but the bladder has not acted; therefore I introduced the catheter. Dressings removed; wound looks well.

16th.—Refreshing sleep in the night. Pulse sixty-eight; pain in the head diminishes daily; breathing easy; bowels soluble; bladder emptied without any assistance. Wound made by the scalpel nearly well. Skin less hot and dry; and the tongue getting cleaner.

Perstat.

17th.—I did not see him; but Mr. Ketchly reports favourably.

Repet^r Mistura et Pilulæ ij.

18th.—Head still easier; pulse sixty-eight; bowels and bladder emptied; hemiplegia undiminished. There is a small hernia cerebri: consequently we increased the pressure and dry lint, with which the wound has heretofore been dressed.

19th.—Going on favourably.

20th.—Pulse sixty, soft, and no longer vibrating. Hernia larger: therefore applied lint moistened with Liquor Calcis, and over it straps and roller.

Repetr Mistura et Pilulæ ij.

21st.—Slept two hours in the night. Pulse sixty; less anxiety, and the patient thinks he shall recover. Hernia less. Skin cool; tongue moist and cleaner.

Repetr Pilulæ ij. Omittr Mistura.

22d.—Passed a comfortable night; pulse sixty. Scalpel wound healed. Bowels and bladder emptied; skin natural, and tongue clean; more sensibility of the left side.

Repetr Pilulæ ij. Dressings the same.

23d.—Slept well; pulse sixty. External wound granulating. A portion of the brain, which was afterwards found to weigh four scruples, being nearly separated, Mr. Ketchly squeezed it off with his fingers, which produced very little pain, and but trifling hemorrhage; and, after its removal, the brain was easily depressed below the cranium.

Dressings the same. Repetr Mistura ut antea.

24th.—The patient passed a comfortable night, is calm, and looks better; pulse sixty-two. There is a good discharge of laudable pus from the surface of the brain, which is now below the cranium at its proper level, and granulations are beginning to rise from its surface; but there is still a small slough to come away. External wound looks well. Pain in left side; bowels open.

25th.—Pulse sixty-four. In every respect going on well.

Repetr Mistura.

27th.—Passed a good night; pulse sixty-six; small. Piece of brain sloughed away; granulations increase. The affected side gets warmer and more sensible.

28th.—Another good night; pulse soft, but quicker; mind tranquil. Wound looks well, and nearly the whole surface of the brain is covered by healthy granulations, a second slough having come away.

Repeat Mixture and Dressing.

29th.—Going on remarkably well; pulse eighty, but not full; bowels well opened.

Omittr Mistura.

30th.—Pulse eighty-two, soft and regular; bowels not acted upon. Surface of the brain covered with granulations, discharge less; external wound looking well, and seems no longer to require keeping open: therefore the lint was discontinued, and the lips of the external wound brought as near together as possible with adhesive straps, and over them a compress and roller to prevent the brain from rising.

Repetr Mistura ut antea.

July 1st.—Slept all night; pulse eighty-two. The brain is inclined to rise, therefore again apply the lint, wet with Liquor Calcis, under the straps. The wound is healing fast, and there is a little motion of the toes of the affected side.

4th.—Going on favourably, but the granulations not quite so healthy. Diminish the pressure.

8th.—Pulse ninety and full, from having indulged his appetite to excess.

℞. Infus. Sennæ ℥j.; Sulph. Mag. ʒvj.; Aq. Menth. ʒx. M. fiat haust. quamprimum sumendus.

11th.—Pulse natural; motion of the inferior extremity restored; arm getting warmer. In every respect going on well. Continues to take broth and gruel, which has been the diet allowed during the progress of the case.

15th.—Granulations being too luxuriant, applied Argent. Nitratum. Inferior extremity gains strength, and the motion of the superior returning.

23d.—Continues getting better and stronger; but, as the wound does not quite heal, it is probable there may be a piece of bone to exfoliate.

August 11th.—Begins to walk upon crutches. A small piece of bone came away the other day. Head nearly well.

23d.—Continues improving. Another fragment of bone came away a few days ago. Wound in the head quite healed; and I discontinued to visit the patient, directing him to be moderate in his diet, and to abstain entirely from malt and spirituous liquors.

This patient went on daily improving, and when I saw him in December last, his health was quite restored. I examined his head, and found that the loss of bone was partially supplied by the usual secretion; and, although I observed a little irregularity in his gait, it perhaps might not have been observed by a person unacquainted with his situation.

*January 13th, 1827.*

